# Solid Microneedles from Poly(3-hydroxybutyrate-co-3-hydroxyvalerate-co-3-hydroxyhexanoate): A Solvent-Free, Biodegradable Platform for Drug Delivery

**DOI:** 10.3390/pharmaceutics18010139

**Published:** 2026-01-22

**Authors:** Diana Araújo, Francisco Santos, Rui Igreja, Filomena Freitas

**Affiliations:** 1 UCIBIO—Applied Molecular Biosciences Unit, Department of Chemistry, School of Science and Technology, NOVA University Lisbon, 2829-516 Caparica, Portugal; fra.santos@campus.fct.unl.pt (F.S.); a4406@fct.unl.pt (F.F.); 2 Associate Laboratory i4HB—Institute for Health and Bioeconomy, School of Science and Technology, NOVA University Lisbon, 2829-516 Caparica, Portugal; 3 CENIMAT/i3N, Department of Materials Science, NOVA School of Science and Technology, NOVA University Lisbon, Campus de Caparica, 2829-516 Caparica, Portugal; rni@fct.unl.pt

**Keywords:** poly(3-hydroxybutyrate-co-3-hydroxyvalerate-co-3-hydroxyhexanoate), solid microneedle (MN) arrays, solvent-free micromolding, coated MNs, transdermal drug delivery

## Abstract

**Background**: Solid microneedles (MNs) are effective transdermal delivery devices but are commonly fabricated from metallic or non-biodegradable materials, raising concerns related to sustainability, waste management, and processing constraints. This study aimed to evaluate the suitability of the biodegradable biopolyester poly(3-hydroxybutyrate-co-3-hydroxyvalerate-co-3-hydroxyhexanoate) (PHBHVHHx) as a structuring material for solvent-free fabrication of solid MN arrays and to assess their mechanical performance, insertion capability, and drug delivery potential. **Methods**: PHBHVHHx MN arrays were fabricated by solvent-free micromolding at 200 °C. The resulting MNs were morphologically characterized by scanning electron microscopy. Mechanical properties were assessed by axial compression testing, and insertion performance was evaluated using a multilayer Parafilm skin simulant model. Diclofenac sodium was used as a model drug and applied via surface coating using a FucoPol-based formulation. In vitro drug release was assessed in phosphate-buffered saline under sink conditions and quantified by UV–Vis spectroscopy. **Results**: PHBHVHHx MN arrays consisted of sharp, well-defined conical needles (681 ± 45 µm length; 330 µm base diameter) with micro-textured surfaces. The MNs withstood compressive forces up to 0.25 ± 0.03 N/needle and achieved insertion depths of approximately 396 µm in the Parafilm model. Drug-coated MNs retained adequate mechanical integrity and exhibited a rapid release profile, with approximately 73% of diclofenac sodium released within 10 min. **Conclusions**: The results demonstrate that PHBHVHHx is a suitable biodegradable thermoplastic for the fabrication of solid MN arrays via a solvent-free process. PHBHVHHx MNs combine adequate mechanical performance, reliable insertion capability, and compatibility with coated drug delivery, supporting their potential as sustainable alternatives to conventional solid MN systems.

## 1. Introduction

Microneedle (MN) technology has emerged as a promising strategy for transdermal drug delivery, enabling painless penetration of the stratum corneum and creating microscopic aqueous pores that allow local or systemic delivery of small molecules, biologics, and vaccines [1,2]. A wide variety of MN types, including solid, coated, dissolving, hollow, and hydrogel-forming systems, have been developed using materials such as silicon, metals, synthetic and natural polymers [3]. Among MN types, solid MNs stand out for their reproducibility, mechanical robustness, compatibility with high-throughput manufacturing, and suitability for post-processing steps (e.g., drug coating or sterilization), making them especially attractive for translation and scalability [4]. Originally designed as skin pretreatment devices, solid MNs have been used as structural supports for coated systems, in which therapeutic agents are deposited on the MNs’ surfaces using coating techniques such as dip-coating, spray coating, or layer-by-layer assembly. These systems enable rapid drug release upon skin insertion while retaining the mechanical advantages of solid MN structures [5].

Despite their great potential, most solid MNs are fabricated from non-biodegradable or mechanically brittle materials, highlighting the need for renewable, biodegradable polymers capable of fabricating robust solid MN structures. Conventional inorganic materials, such as silicon, metals, and ceramics, are brittle and non-degradable, whereas many synthetic polymers require organic solvents, raising regulatory and safety challenges [6]. Natural polymers, including chitosan, cellulose, hyaluronic acid, and alginate, are biocompatible and biodegradable, and have been widely used for the fabrication of dissolving or hydrogel MNs [7].

Among natural polymers, polyhydroxyalkanoates (PHAs) represent a promising class of biodegradable thermoplastics due to their mechanical tunability, biocompatibility, and thermal processability. These bacterial polyesters exhibit mechanical properties ranging from rigid and brittle short-chain-length PHAs (scl-PHAs) (e.g., poly(3-hydroxybutyrate), PHB) to flexible and elastic medium-chain length PHAs (mcl-PHAs), depending on the monomers’ chain length [8,9]. Copolymers incorporating both scl and mcl monomers combine the high tensile strength characteristic of scl-PHAs with the flexibility of mcl-PHA polymers. The terpolymer poly(3-hydroxybutyrate-co-3-hydroxyvalerate-co-3-hydroxyhexanoate) (PHBHVHHx) is one such material, distinguished by its ductility and lower crystallinity [10,11,12,13], enabling micromolding without brittle fracture. These interesting mechanical properties, alongside the broader advantages of PHAs, make PHBHVHHx a particularly strong candidate for solvent-free fabrication of solid MNs. Only recently have PHAs been explored for MN fabrication. The first reported PHA-based MN arrays used poly(3-hydroxybutyrate-co-3-hydroxyvalerate) (PHBHV) with a 3HV content of 21% by a thermosetting process, yielding conical MNs with good mechanical properties (0.27 N/needle) and the ability to be impregnated with the fluorescent dye rhodamine 6G [14].

Among all polymeric MN fabrication processes, the micromolding technique has been the most widely applied, as it offers good reproducibility and cost-effectiveness [15]. Conventional micromolding typically relies on polymer dissolution in a suitable solvent that must be cast and dried within microstructured molds [16], which can require organic solvents for certain polymers [17]. For thermoplastic polymers with high thermal stability, solvent-free micromolding, which processes the polymer in its molten state, offers a safer and more sustainable alternative. This strategy was tested for the fabrication of MNs based on polycaprolactone (PCL) [17], polylactic acid (PLA) [18], and poly(lactic-co-glycolic) acid (PLGA) [19].

In this study, the terpolymer PHBHVHHx was used as a structural biomaterial for the fabrication of solid MN arrays through a solvent-free micromolding process. The resulting MNs were characterized in terms of morphology, mechanical properties, and insertion capability. Moreover, diclofenac sodium was coated onto the MN surface to evaluate the feasibility of PHBHVHHx-based MNs as a biodegradable and sustainable platform for transdermal drug delivery.

## 2. Materials and Methods

### 2.1. Biopolymer Production and Characterization

The terpolymer PHBHVHHx was produced by a mixed microbial culture using fermented fruit pulp as the carbon source, as previously described [20]. The biopolymer was recovered from the freeze-dried biomass using Soxhlet extraction with chloroform, followed by precipitation in ice-cold ethanol and subsequent drying, as described by Pereira et al. [11]. The thermal behavior of PHBHVHHx was evaluated by thermogravimetric analysis (TGA) using a thermogravimetric analyzer (Setaram Labsys EVO, Sophia Antipolis, France). For the dynamic TGA, biopolymer samples (~14 mg) were placed in aluminum crucibles and heated from 25 to 500 °C at a heating rate of 10 °C/min under an argon atmosphere. The thermal stability of PHBHVHHx was further assessed by isothermal TGA, in which samples were heated to 200 °C and maintained at this temperature for 120 min under an argon atmosphere. All experiments reported in this study were performed using PHBHVHHx obtained from a single production batch.

FucoPol was obtained by cultivation of the bacterium *Enterobacter* A47 (DSM 23139) in a 10 L bioreactor (BioStat B-plus, Sartorius, Gottingen, Germany) using glycerol as the sole carbon source, as described by Concórdio-Reis et al. [21]. The biopolymer was recovered and purified from the cultivation broth via diafiltration and ultrafiltration, as previously described [22] and characterized as reported by Araújo et al. [23]. FucoPol used in this study was composed of 36 mol% fucose, 33 mol% glucose, 26 mol% galactose, 5 mol% glucuronic acid, and 7.8 wt% of acyl groups, with an average molecular weight (Mw) of 3.2 × 10^6^ Da and a polydispersity index (PDI) of 1.9.

### 2.2. Preparation of the PHBHVHHx MN Arrays

The PHBHVHHx MN arrays were prepared by a solvent-free hot micromolding technique, using polydimethylsiloxane (PDMS) molds produced as previously described [14]. Briefly, PDMS sheets were fabricated by mixing PDMS pre-polymer (Silicone Elastomer, Sylgard 184, Dow Corning, Midland, MI, USA) and curing agent at a 10:1 (*v*/*v*) ratio, casting the mixture into Petri dishes and degassing under vacuum, and then curing at 70 °C for 1 h. The sheets were subsequently used to fabricate molds by engraving controlled microstructures with a pulsed CO_2_ laser system (Universal Laser System, VLS3.5, Vienna, Austria). Laser engraving was performed using a 2.0” lens with a laser power of 30 W and a speed of 0.15 m/s to produce a spiral pattern. The final PDMS molds comprised an array of 15 × 15 MNs, in an area of 1 cm^2^, where each conical spiral microcavity has a length of approximately 690 µm, a base diameter of 330 µm, and a pitch of 600 µm.

PHBHVHHx MN arrays were produced through an adaptation of the procedure described by Silvestre et al. [14]. The biopolymer (0.1 g) was placed over the PDMS mold and subjected to a heat treatment comprising three main steps: first, the temperature was gradually increased from room temperature to 200 °C, for 20 min; second, the temperature was maintained at 200 °C for 20 min; then, a weight (~40 g) was placed on top of the melted biopolymer to promote the filling of the microcavities as the temperature decreased until room temperature. The processing temperature of 200 °C was selected to ensure sufficient softening and flow of the molten PHBHVHHx for complete filling of the PDMS microcavities, while remaining below the onset of thermal degradation. Finally, the sample was removed from the oven, the demolding process was performed, and the PHBHVHHx MN array was obtained.

### 2.3. MN Arrays’ Characterization

#### 2.3.1. Morphological Characterization

The morphology of the PHBHVHHx MNs was assessed by scanning electron microscopy (SEM). MN samples were coated with gold-palladium (~20 nm) and analyzed using a bench scanning electron microscope (TM3030Plus + Quantax 70, Hitachi High Technologies, Tokyo, Japan) with an acceleration voltage of 15 kV. MN dimensions were determined by SEM observations using ImageJ version 1.53k (National Institutes of Health, Bethesda, MD, USA). The morphology of the MNs after applying a compression force of 0.058 N/needle was further examined using a stereo microscope (M80, Leica Microsystems, Wetzlar, Germany).

#### 2.3.2. Compressive Axial Mechanical Analysis

The mechanical axial compressive properties of the PHBHVHHx MNs were assessed using a Precision Universal Testing Machine (Shimadzu, Autograph GX-V2, Kyoto, Japan) equipped with a 1 kN load cell. Compression tests were performed on complete MN arrays placed on the base plate and a flat stainless-steel platen with a diameter of 10 cm. An axial force at a constant speed of 0.05 mm/s was applied. The test was performed with a trigger force of 0.1 N, under ambient conditions (20 °C, 50% humidity).

#### 2.3.3. Insertion Studies

Insertion performance was evaluated using a commercial polymeric film (Parafilm^®^, Bemis Company Inc., Soignies, Belgium) as a model membrane. To simulate the thickness of excised skin (~1 mm), a sheet of Parafilm was folded into eight layers, as described by Larrañeta et al. [24], and the thickness of the Parafilm sheet was measured using a micrometer (Elcometer, Manchester, UK). MN arrays were inserted into the Parafilm surface using a texture analyzer in compression mode, by applying a force of 40 N, for 30 s. Then, each Parafilm layer was examined under the stereo microscope, and the number of perforations was recorded.

### 2.4. Preparation of Coated MNs

#### 2.4.1. Coating of the PHBHVHHx MNs

**Figure 1:**
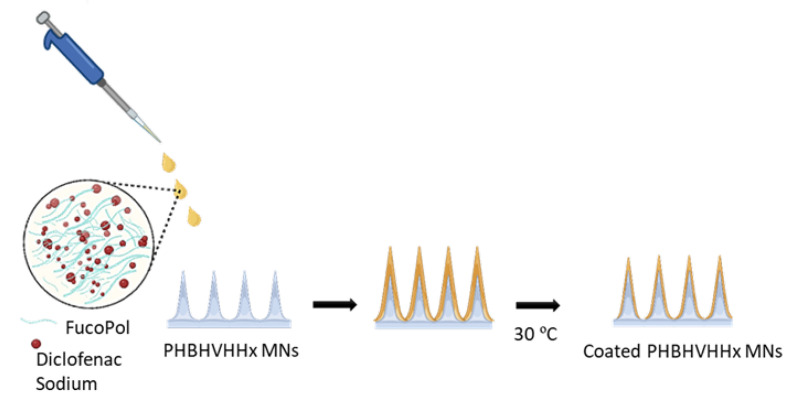
Schematic illustration of the preparation of diclofenac sodium-coated PHBHVHHx MNs.

PHBHVHHx MN arrays were coated with diclofenac sodium (98%, Tokyo Chemical Industry Co., Tokyo, Japan) using a simple drop-coating method. For fabricating the coated MNs, 200 µL aliquots of an aqueous solution of FucoPol (1.0%, *w*/*v*) and diclofenac sodium (1.0, *w*/*v*) were dropped on the surface of the PHBHVHHx MNs using a micropipette (Figure 1), ensuring even distribution of the coating solution across the array. FucoPol was used as a thickening agent to increase the viscosity of the solution and, hence, promote adherence onto the needles’ surface. MN arrays were dried in an oven at 30 °C under static air conditions until complete solvent evaporation was visually confirmed. All coating experiments were performed on at least three independently prepared MN arrays. The drug content of the coated MN arrays was determined gravimetrically, assuming diclofenac sodium accounted for 50% (*w*/*w*) of the FucoPol-diclofenac coating formulation, based on the mass difference between coated and uncoated arrays.

#### 2.4.2. In Vitro Drug Release Studies

Drug release studies were performed by immersing the coated MN arrays in 10 mL of phosphate-buffered saline (PBS, pH 7.4) at 37 °C, under constant magnetic stirring (50 rpm). At predetermined time intervals, 1 mL samples of the release medium were withdrawn and replaced with an equal volume of preheated PBS. This procedure ensured that sink conditions were maintained throughout the experiment, with the drug concentration remaining below saturation. The diclofenac concentration in the withdrawn samples was determined using a UV-Vis spectrophotometer (CamSpec M509T, Leeds, UK) at a wavelength of 275 nm [25].

## 3. Results and Discussion

### 3.1. Biopolymer Characterization

The PHBHVHHx terpolymer used in this study was composed of 55 wt% 3HB, 21 wt% 3HV, and 24 wt% 3HHx, with an average Mw of 0.9 × 10^5^ Da, and a PDI of 2.2 [11]. It presented a Young’s modulus of 78.3 ± 6.9 MPa, a tensile strength of 5.1 ± 0.2 MPa, and an elongation at break of 269.2 ± 52.1%, indicating a material with moderate stiffness and high ductility. The terpolymer exhibited an isomorphism phenomenon, presenting two melting temperatures at 144 °C and 159 °C, which can be attributed to the high contents of the 3HV and 3HHx monomers (21 and 24 wt%, respectively) [11]. It displayed a glass transition temperature of −3.8 °C and a crystallinity index of 26.2%.

**Figure 2:**
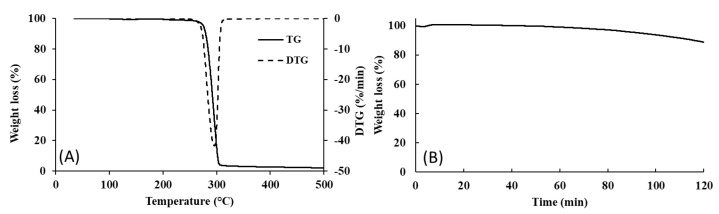
Thermogravimetric curve (**A**) and isothermal TGA (**B**) of the terpolymer PHBHVHHx.

As shown in the TGA curve (Figure 2A), the biopolymer is thermally stable up to approximately 260 °C, undergoing rapid degradation in a single-step process. A weight loss of 5% occurs at 276 °C, with a maximum degradation rate between 275 °C and 315 °C, where a major weight loss was observed (92%). Isothermal TGA analysis performed at 200 °C for 120 min revealed that no weight loss was observed within the first 40 min, and only a minor weight loss of 5% was noticed after 94 min of exposure (Figure 2B). After 120 min, around 89% of the initial mass was still retained, thus showing the biopolymer’s thermal stability, enduring a long period of exposure to a temperature of 200 °C with minor degradation. A similar behavior was reported for PHB, which displayed a weight loss of approximately 2% after 50 min at 200 °C, under a nitrogen atmosphere [26]. Xiang et al. [27] also reported that a PHBHV copolymer with a 3HV content of 3% suffered no significant mass changes upon exposure to a temperature of 210 °C for 30 min.

The thermal and mechanical properties, as well as the crystallinity of the biopolymer, are crucial for its use in the fabrication of MNs. Its high thermal stability makes it suitable for processing at 200 °C with no degradation, while maintaining its integrity during the MN fabrication process. The biopolymer exhibits moderate stiffness and tensile strength, which can provide sufficient rigidity to penetrate the skin, with enough flexibility (low crystallinity index and low Young’s modulus) to prevent brittleness. These properties will help the MNs absorb mechanical stress without fracturing, thus making the PHBHVHHx terpolymer well-suited for the fabrication of solid MN arrays.

### 3.2. Fabrication of the PHBHVHHx MN Arrays

**Figure 3:**
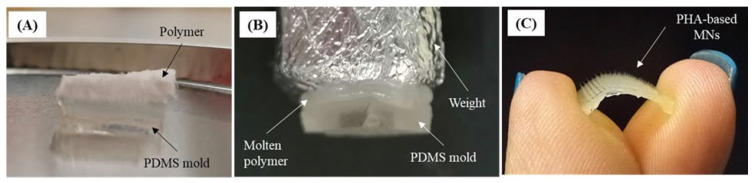
Three-step process for the MN array fabrication: (**A**) PHBHVHHx terpolymer sample over the PDMS mold before being submitted to a heat treatment at 200 °C, (**B**) filling the mold microcavities with molten polymer, and (**C**) demolding process and obtaining the MN array.

The thermal stability and melt-processing behavior of PHBHVHHx enable its successful fabrication into MN arrays using a solvent-free micromolding process adapted from Silvestre et al. [14]. As shown in Figure 3, fabrication consisted of three sequential steps. First, the polymer was placed over the PDMS mold (Figure 3A), and upon heating, it transitioned into a molten state and gradually filled the conical microcavities under the applied load (Figure 3B). After cooling, the solidified MN array was easily demolded, yielding a flexible and uniform structure (Figure 3C).

During processing, the polymer softened homogenously and flowed efficiently into the PDMS mold microcavities, replicating their geometry with high reproducibility. The terpolymer exhibited high moldability, producing well-defined and sharply tipped MNs across the entire array. The intact demolding of the structures further confirmed that PHBHVHHx possesses an adequate combination of melt viscosity, ductility, and mechanical integrity for solvent-free micromolding fabrication. This process consistently resulted in a high fabrication yield, with more than 90% of MN arrays per batch being successfully demolded without structural defects. These results demonstrate that the terpolymer can be processed into high-resolution MN structures using a simple, scalable, and environmentally conscious manufacturing approach.

From a translational manufacturing perspective, the thermoplastic nature and high-temperature processing tolerance of PHBHVHHx also suggest compatibility with common low-temperature sterilization methods (e.g., ethylene oxide or gamma irradiation), although the impact of sterilization on MN performance and coating integrity will require evaluation in future studies.

### 3.3. Morphological Characterization

**Figure 4:**
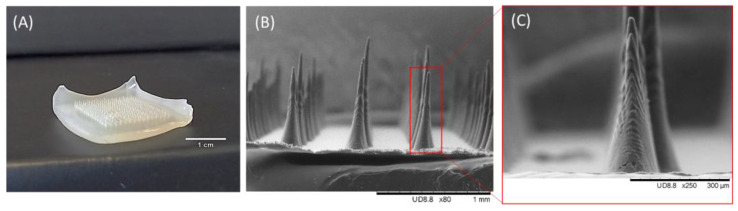
Photograph (**A**) and SEM images (**B**,**C**) of PHBHVHHx MN arrays. The MN image observed under magnification 80× (**B**) was expanded to observe a single MN under magnification 250× (**C**).

As depicted in Figure 4, the PHBHVHHx MNs reproduced the mold design with high reproducibility. Each array comprised a total of 225 MNs, arranged in a 15 × 15 square configuration, with a total area of 1 cm^2^. The resulting arrays retained the white and translucent appearance of the original polymer, evidencing that no thermal degradation occurred during processing (Figure 4A). The MNs exhibited a uniform conical geometry (Figure 4B), with well-defined and sharp tips. The average MN length was 681 ± 45 µm, with a base diameter of approximately 330 µm, concomitant with the PDMS mold dimensions. These lengths are within the commonly reported range for MNs (150–1500 µm) [28], and heights above 600 µm are particularly advantageous for skin penetration [29]. SEM images of multiple independently fabricated arrays revealed that all MNs were fully formed, with no visible defects such as incomplete filling, broken tips, or missing needles, indicating high reproducibility of the micromolding process. SEM analysis also revealed a micro-textured surface characterized by ridges and valleys originating from the mold’s laser spiral pattern (Figure 4C). Such surface topography is expected to benefit coating-based strategies by promoting a more homogeneous distribution of the coating formulation along the height of the MN rather than accumulation at the tip. Furthermore, the increased surface area may enhance drug loading capacity, potentially increasing the MNs’ capacity to carry and deliver higher amounts of therapeutic agents [30].

### 3.4. Mechanical Characterization of the MNs

**Figure 5:**
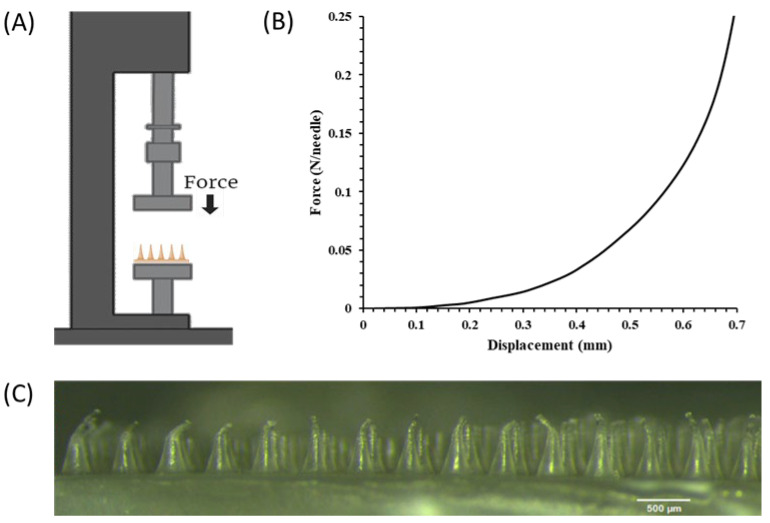
Mechanical characterization of PHBHVHHx MN arrays: (**A**) schematic diagram of compression mechanical tester; (**B**) compression stress–strain curves of the MN arrays; (**C**) optical image of PHBHVHHx MN array after compression (0.058 N/needle).

The ability of MNs to safely and efficiently deliver the loaded drugs is dependent on their mechanical strength being greater than the force required to pierce the skin [31]. As such, the mechanical properties of the fabricated MN arrays were evaluated by a compression test, where the axial force loaded vs. the displacement behavior was assessed using a texture analyzer, as illustrated in Figure 5A.

The force–displacement curves of the PHBHVHHx MN arrays (Figure 5B) showed a non-linear increase in axial compressive force up to 0.25 ± 0.03 N/needle at a displacement of 0.68 mm (equivalent to the nominal MN length). Although the typical sudden drop in force associated with MN failure was not present, the MNs were visibly deformed after the test, indicating that the failure force was below the maximum determined. This lack of a clear failure point is consistent with previous studies of thermoplastic polymer MNs and may indicate (i) a gradual compression of the MNs without buckling or (ii) that the MNs in the array did not all fail simultaneously [32,33]. Ando et al. [31] reported that PVA MNs with a high aspect ratio (2.8) show local maximum points in the force–displacement curves, demonstrating buckling deformation, whereas those with a smaller aspect ratio (1.8) show a progressive deformation, starting near the tip and moving downward with increasing force, but never demonstrating a catastrophic buckling event at a single point of failure. On the other hand, several studies have suggested that, when testing whole MN arrays, even if some MNs fail by sudden buckling, the failure point of any individual MN can be masked by the collective strength of the other MNs (if the MNs fail at different times) [32,34,35]. This compressive response, together with the observation that the PHBHVHHx MNs were crushed, without fracture, after the compression tests, suggests these MNs undergo progressive plastic deformation/bending rather than brittle catastrophic failure [17]. This behavior can be advantageous as it reduces the risk of tip breakage and potential fragment retention in the skin. However, MNs may still bend or twist during insertion, due to differences in skin thickness and elasticity as well as insertion speed, non-uniform contact, and off-axis loading, potentially affecting the efficiency of MN insertion even in the absence of fracture [34].

Previously reported MN arrays prepared with PHBHV with a 21 wt% 3HV content [14] showed a similar force–displacement behavior, with a maximum force of between 0.27 and 0.69 N/needle, depending on the MN pitch. The slightly lower value obtained here may be explained by the different monomer composition of the PHA used to prepare the MN arrays. The terpolymer used in this study had a 3HHx content of 24 wt% in its composition, which leads to a decrease in crystallinity and an increase in chain flexibility, contributing to a lower mechanical strength [10].

The mechanical strength of solid polymeric MNs reported varies significantly depending on several factors, such as the intrinsic properties of the materials used for fabrication, the geometry and dimensions of the individual MNs, and the MN pitch, height variability, and composition heterogeneity in whole MN arrays [32,36]. Shah et al. [37] reported failure force values of ~0.065 and ~0.078 N/needle for MNs produced using dextran and hyaluronic acid, respectively. On the other hand, higher failure forces were observed for MNs prepared with PVA (0.32 ± 0.07 N/needle) [32] and sodium alginate (0.18 ± 0.05 N/needle). In the context of biodegradable thermoplastic-based MNs, PHBHVHHx MNs exhibited a force–displacement response comparable to that reported for PCL MNs, while achieving a higher maximum force (0.25 vs. 0.16 N/needle), which may indicate improved mechanical robustness during handling and insertion [17]. In comparison, MNs fabricated from PLGA have been reported to exhibit significantly higher mechanical resistance to axial load, with a maximum force of 1.06 ± 0.02 N/needle [38]. Nevertheless, it has been widely reported that the minimum insertion force required for MNs to puncture skin is 0.058 N/needle, where MNs capable of withstanding are considered suitable for use in drug delivery [39]. Therefore, to confirm the adequate mechanical performance of the PHBHVHHx MNs fabricated, the MN array was subjected to 0.058 N/needle for 10 s using the same setup as the compression tests and evaluated by morphological analysis. As can be seen in Figure 5C, the tip of the MN structure was slightly bent, but without significant deformation of the overall structure, indicating that although some elastic or plastic deformation may occur, the fabricated MN arrays retain sufficient structural integrity for reliable human skin penetration. This deformation-dominated response further indicates that PHBHVHHx MNs are best suited for single-use applications, as repeated insertion cycles could progressively increase tip bending.

### 3.5. Insertion Studies

**Figure 6:**
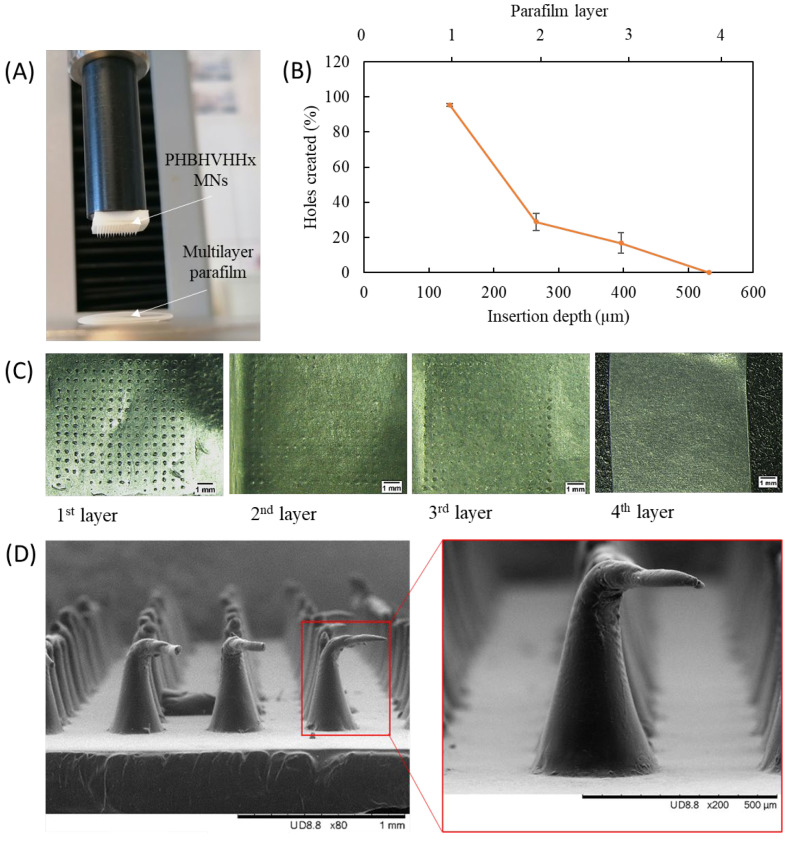
Parafilm insertion studies: (**A**) optical image of the setup; (**B**) percentage of holes created in each Parafilm layer using PHBHVHHx MNs; (**C**) optical images of Parafilm layers after insertion; (**D**) SEM images of PHBHVHHx-based MNs after insertion in Parafilm.

The insertion ability of the PHBHVHHx MN arrays was assessed using an eight-layer Parafilm model system, which mimics the approximately 1 mm thickness of excised skin (Figure 6) [24]. Prior to testing, the thickness of an individual Parafilm layer was measured and determined to be 132 µm, which was used to estimate insertion depth. The arrays were positioned on top of the Parafilm multilayer, and an axial force of 40 N (reported as the maximum average force applied manually [24]) was applied for 30 s (Figure 6A). The number of perforations produced in each layer is presented in Figure 6B.

The first layer of parafilm, corresponding to a depth of 132 µm, was almost fully pierced (95.3 ± 0.8%), while only a small number of MNs reached the second (28.9 ± 4.9%) and the third layers (16.9 ± 5.8%). Moreover, none of the MNs were able to penetrate the fourth layer of the parafilm model. While these results reflect the average insertion performance of the MN arrays, variability in penetration among individual MNs within the same array was not quantitatively analyzed. An identical result was obtained for hyaluronic acid-based MNs that fully pierced the first layer of parafilm, but only 72% and 19% of the MNs could pierce the second and third layers, respectively [40]. Silva et al. [25] also demonstrated the ability of carboxymethylcellulose (CMC) MNs to penetrate the first layer completely, decreasing the percentage of puncture to 90% for the second layer and to 29% for the third layer. In contrast, commercial stainless steel MN devices such as Dermapen^®^ and Dermastamp™ exhibit higher insertion efficiency, with approximately 100% penetration of the first three layers and partial penetration (<50%) of the fifth and sixth layers [41]. Differences in penetration efficiency can be partly explained by MN geometry, as the structures differ in aspect ratio, needle length, and tip radius. Hyaluronic acid-based MNs [40] and CMC-based MNs [25] presented heights around 460 µm and base widths of 200 µm, with aspect ratios close to 2.3. On the other hand, commercial devices rely on longer needles (~1 mm) with larger and more variable tip radii [41], geometrical parameters known to influence insertion force and penetration depth in Parafilm models.

The results demonstrated that PHBHVHHx MNs consistently pierced the first parafilm layer and reached the third layer, corresponding to a thickness of around 396 µm (Figure 6C). This performance suggests that these MNs would readily traverse the stratum corneum (~10 to 30 µm of thickness), allowing insertion into the viable epidermis [34,42]. Given the MNs’ average height (681 ± 45 µm) and pierced depth (~396 µm), the penetration achieved represents approximately 58% of the MNs’ length. This result is consistent with previous studies indicating that Parafilm penetration often overestimates insertion depth relative to biological tissue. Taken together, these studies establish an empirical correlation between Parafilm layer penetration and effective skin insertion depth, supporting its use as a predictive screening model for the performance of MN insertion [24]. For instance, Fonseca et al. [40] developed a patch of rutin-loaded hyaluronic acid/bacterial cellulose MNs able to pierce 81% of the third parafilm layer, and insertion tests into porcine ear skin revealed their capacity to create cavities with 47.3–99.3 µm of depth that correspond to ~22% of the height of the MNs. Similarly, a patch of CMC-based MNs loaded with diclofenac sodium that pierced 99%, 86%, and 18% of the first, second, and third parafilm layers, respectively, demonstrated the ability to puncture ex vivo human skin with depths of 133–401 µm, equivalent to 29% and 88% of the MNs’ height [25]. The similarities of the results reinforce that the PHBHVHHx MNs are expected to create microchannels in the skin, allowing the transdermal delivery of molecules.

As shown in Figure 6D, following insertion, the MNs remained unbroken, exhibiting a slight bending of the tip without any damage to the MN’s structure. This behavior is consistent with that reported for other polymeric MN systems, such as CMC [25] and sodium alginate MNs [43]. These findings confirm that PHBHVHHx MNs withstand the forces required for skin penetration without mechanical failure. PHBHVHHx is a biodegradable microbial polyester whose degradation occurs mainly through enzymatic hydrolysis and is typically governed by surface erosion rather than bulk degradation. In this application, solid PHBHVHHx MNs are designed to act as transient mechanical carriers and are removed after insertion, rather than degrading within the skin. This means that degradation is not expected to occur during the short insertion timeframe, resulting in minimal polymer retention in the tissue. Compared with conventional hypodermic injections, PHBHVHHx-based delivery eliminates the use of metallic needles and sharps, reducing biohazardous waste. Additionally, PHAs degrade into naturally occurring hydroxyalkanoic acids that enter endogenous metabolic pathways, and PHA-based materials have demonstrated good biocompatibility in contact with biological tissues [44].

### 3.6. PHBHVHHx MNs Loaded with Diclofenac Sodium

PHBHVHHx MN arrays were coated using a drop coating method with diclofenac sodium as a model drug. FucoPol was incorporated into the coating formulation as a thickening agent to improve the viscosity of the solution [45], thus improving its adhesion to the MN surfaces. Moreover, FucoPol’s potential bioadhesive or skin-interacting properties may enhance coating retention on the MN surface and modulate drug–skin interactions following insertion, potentially affecting in vivo drug delivery behavior. This strategy aligns with previous reports in which thickening polymers were used to enhance coating efficiency. For instance, PLA-based MNs coated with sulforhodamine B employed polyvinyl alcohol (PVA) to control coating solution viscosity, and increasing the viscosity from 150 to 20,100 mPa·s resulted in higher drug loading from 2.5 ng/needle to 33.4 ng/needle [46]. Similarly, Wu et al. [47] also reported the preparation of identical rhodamine B-coated PLA MNs, using CMC as a viscosity modifier.

**Figure 7:**
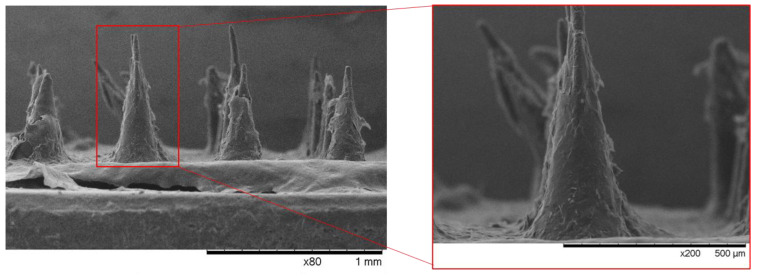
SEM images of diclofenac-coated PHBHVHHx MN arrays prepared using a FucoPol-thickened drop-coating solution to ensure uniform drug deposition.

Using this approach, PHBHVHHx MNs were successfully coated, achieving a drug loading of 1.57 ± 0.37 mg of diclofenac/array. As shown in Figure 7, the diclofenac layer adhered uniformly along the entire length of the PHBHVHHx MNs. At low magnification, the coated arrays maintained their structural integrity, and the base of the array remained smooth and continuous. Higher magnification imaging revealed an irregular but continuous layer surrounding each MN. However, no quantitative assessment of coating thickness distribution along the MN length was performed, and potential local variations in coating thickness along the tip–shaft axis cannot be excluded. The coated MN arrays were subsequently characterized for their mechanical properties and drug release studies.

#### 3.6.1. Mechanical Properties

The compressive force-displacement curves of the diclofenac sodium-coated MN arrays showed a similar behavior, with no clear failure point. However, the force at a displacement equivalent to the nominal MN length increased from 0.25 ± 0.03 N/needle for the uncoated samples to 0.37 ± 0.13 N/needle. As such, based on the results from the uncoated samples, the diclofenac sodium-coated MNs also possess the mechanical properties required to pierce the skin, evidencing their possible use as transdermal delivery systems.

The increase in mechanical strength observed for the coated samples can be attributed to FucoPol, which may provide some mechanical reinforcement to the MN when dried. A similar increase in mechanical stiffness was reported for the acrylic-based MNs upon coating with polypyrrole [48], for which a force of 0.08 N/needle led to a displacement of 48.1 ± 8.79 µm for the uncoated MNs, while a lower displacement value (33.7 ± 4.09 µm) was obtained for the polypyrrole-coated MNs. This increase is also observed in porous PLGA MNs, where a coating of CMC improved compression fracture from around 0.006 N/needle (uncoated MN) to over 0.07 N/needle. On the other hand, the mechanical properties of MNs coated with small molecules (e.g., sulforhodamine B) have been reported to show no significant difference from the uncoated MNs [46].

#### 3.6.2. In Vitro Drug Release Studies

**Figure 8:**
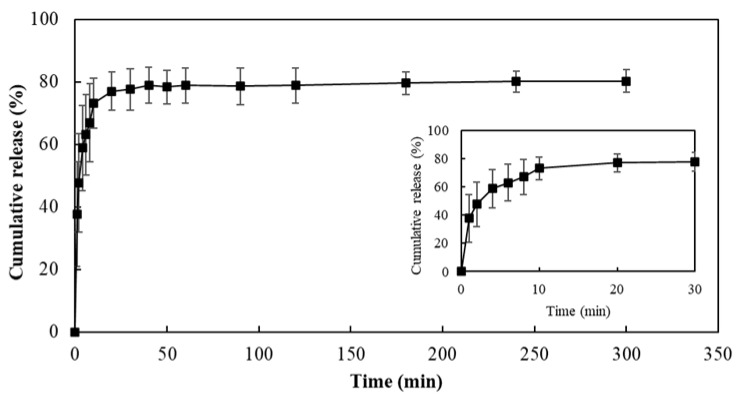
Cumulative release profile of diclofenac from coated PHBHVHHx MNs. The inset graph highlights the initial fast release.

As shown in Figure 8, the release profile of diclofenac sodium from the PHBHVHHx MN arrays, evaluated in PBS at 37 °C, was characterized by an initial burst release followed by a slower release phase. Within the first 10 min, 73% of the loaded drug was released, after which the release rate progressively decreased, reaching a plateau between 10 and 40 min. After 300 min, the cumulative release reached 80%.

The initial burst phase is attributed to the diclofenac deposited on the MN surface, which rapidly dissolves upon contact with the aqueous medium. Comparable burst-type release profiles have been reported for other coated MN systems. For instance, bioceramics-based MNs coated with ovalbumin released 43% of the drug within 10 min, and up to 90% was achieved after 60 min in PBS at room temperature [49]. Zhou and co-workers [50] prepared PLA MNs coated with bovine serum albumin (BSA), and under similar in vitro release conditions, around 40% of BSA was released within the first 24 h. Considering the therapeutic application of diclofenac sodium, this rapid-release profile is desirable, as it enables immediate local analgesic and anti-inflammatory action [51]. Compared with commercially available topical diclofenac formulations, which rely on passive diffusion across the stratum corneum, the coated MN approach enables rapid drug deposition directly within the viable epidermis and upper dermis. Therefore, the observed burst release reflects a functional advantage of this delivery strategy rather than a limitation. However, it should be noted that in vitro release profiles represent a worst-case scenario for release rate, as drug diffusion and clearance in skin are expected to moderate the effective release kinetics in vivo [52]. The release mechanism of diclofenac sodium from the coated MN arrays was investigated by fitting the first 60% of the release data to the Korsmeyer–Peppas model [53]. The release profile showed an excellent fit to the model (R^2^ = 0.99), and the obtained release exponent (*n* = 0.319) indicates that drug release follows a Fickian diffusion mechanism, suggesting that release is predominantly governed by diffusion through the hydrated FucoPol coating rather than bulk polymer erosion. The release profile confirms that PHBHVHHx MNs can serve as an efficient platform for delivering fast-acting drugs through a coated solid MN strategy.

Despite providing robust and reproducible evidence of PHBHVHHx MNs’ fabrication quality, mechanical integrity, and insertion capability, this study has limitations, including the use of Parafilm as a skin simulant rather than biological skin, the lack of long-term stability assessment, and the absence of cytotoxicity or biocompatibility studies. Building on these results, future work will focus on validating PHBHVHHx solid MNs in biologically relevant skin models, refining coating formulations and loading strategies to modulate drug release behavior, and expanding the application of this platform to other low-dose therapeutics.

## 4. Conclusions

This study demonstrated the successful preparation of MN arrays based on the biopolyester PHBHVHHx, through a solvent-free micromolding process. The obtained MNs exhibited conical geometries, sharp tips, and micro-textured surfaces, enabling reproducible replication of the mold design. Mechanical testing and insertion studies confirmed that the MNs possessed sufficient strength to penetrate up to the third layer of the parafilm skin model, indicating their suitability for breaching the stratum corneum and reaching the viable epidermis. Coating the MN arrays with diclofenac sodium using a FucoPol-thickened formulation resulted in uniform drug deposition without compromising structural integrity. The coated MNs maintained adequate mechanical performance for skin insertion and rapidly released diclofenac in vitro, achieving a burst release profile consistent with the therapeutic need for rapid onset of analgesic and anti-inflammatory action. Overall, these findings demonstrate that the PHBHVHHx terpolymer is a promising biomaterial for the development of biodegradable, solvent-free, and minimally invasive platforms, supporting its potential for future applications in transdermal drug delivery.

## Data Availability

The original contributions presented in this study are included in the article. Further inquiries can be directed to the corresponding author.

## Abbreviations

PHBHVHHx	Poly(3-hydroxybutyrate-co-3-hydroxyvalerate-co-3-hydroxyhexanoate)
MN	Microneedle
PHAs	Polyhydroxyalkanoates
Scl-PHAs	Short-chain-length PHAs
Mcl-PHAs	Medium-chain length PHAs
PHBHV	Poly(3-hydroxybutyrate-co-3-hydroxyvalerate)
PCL	Polycaprolactone
PLA	Polylactic acid
PLGA	Poly(lactic-co-glycolic) acid
TGA	Thermogravimetric analysis
Mw	Average molecular weight
PDI	Polydispersity index
PDMS	Polydimethylsiloxane
SEM	Scanning electron microscopy
PBS	Phosphate-buffered saline
CMC	Carboxymethylcellulose
PVA	Polyvinyl alcohol
BSA	Bovine serum albumin
